# Relationships between dimensions of fatigue and psychological distress among public hospital nurses

**DOI:** 10.15171/hpp.2018.26

**Published:** 2018-07-07

**Authors:** Ahmad Bazazan, Iman Dianat, Leila Rastgoo, Hojat Zandi

**Affiliations:** ^1^Department of Occupational Health and Ergonomics, Tabriz University of Medical Sciences, Tabriz, Iran; ^2^Department of Nursing, Shahid Beheshti University of Medical Sciences, Tehran, Iran; ^3^Department of Health Safety and Environment (HSE), Shahid Beheshti University of Medical Sciences, Tehran, Iran

**Keywords:** Mental distress, Health care, Mental disorders, Mental health, Nursing

## Abstract

**Background:** Fatigue and psychological distress are fairly common among nursing personnel,but their relationship is not clearly understood.

**Methods:** In this cross-sectional study, we investigated the relationships between dimensions of fatigue and psychological distress among 699 public hospital nurses in Iran. The Multidimensional Fatigue Inventory (MFI–20) and General Health Questionnaire (GHQ–12) were used for evaluation of fatigue and psychological distress, respectively.

**Results:** The total fatigue score of the MFI–20 was 52.5 (SD = 11.8). The general fatigue (mean± SD = 13.2±3.1) and reduced motivation (mean ± SD = 9.2±2.7) were the fatigue dimensions with the highest and lowest mean scores, respectively. The percentage of psychological distress(e.g., those nurses scored above the cut-off point [GHQ–12 ≥ 4]) was 29.1%. Stepwise multiple linear regression analysis indicated that general (P<0.001), physical (P<0.001) and mental (P<0.001) aspects of fatigue were significantly related to the psychological distress, so that the scores of these fatigue dimensions were significantly higher for those nurses with a higher level of psychological distress (above the cut-off point) than those with a lower level of psychological distress (below the cut-off point).

**Conclusion:** The results emphasize the need for multi-component interventions aimed at decreasing both mental and physical fatigue, and consequently reducing the psychological distress among this group of healthcare professionals.

## Introduction

Nurses constitute the largest group of healthcare professionals in almost all countries. Working conditions of hospital nurses impose high levels of mental and physical demands on this group of healthcare professionals.^1-3^ Previous research has shown that nurses may experience a higher level of fatigue compared to other healthcare professionals.^4-7^ According to the literature, fatigue has significant consequences in terms of health and safety, job satisfaction, job performance, stress, burnout and absent from work among nurses.^8-11^ Fatigue in nurses may also have serious implications in terms of patient safety and quality of health care.^12^

There is also evidence that psychological distress is also a common problem among nurses.^1,13^ This may be attributed to stressful working conditions in hospitals and clinical settings and to the fact that nurses have to continuously develop their new skills to cope with advances in their job.^1^ A better understanding the of the factors associated with psychological distress among nurses not only has implications in terms of health promotion of this working group, but also in the quality of care provided.

To the authors’ knowledge, the relationship of fatigue as a multidimensional phenomenon and psychological distress has not been explored sufficiently in hospital nurses. Therefore, in an attempt to address this issue, this cross-sectionai study was conducted to evaluate the relationship between multiple dimensions of fatigue and psychological distress among a sample of public hospital nurses in Iran.

## Materials and Methods

### Study design, procedure and sample 

This study was cross-sectional in design and was carried out in two public hospitals in Tehran (the capital of Iran). Data on socio-demographic and job details, fatigue and psychological distress of hospital nurses were collected using a questionnaire. All nurses who worked for at least one year in their current job and had no physical disability or mental disease (assessed by self-report) were asked to take part in the study. Sample selection was based on convenience sampling. For this, a total of 949 questionnaires were distributed among eligible participants, of which 699 nurses completed and returned the questionnaires. This represented a response rate of 73.6%. All volunteered nurses were familiarized with the study and signed a written informed consent form before participation.

### Measures 

*Socio-demographic and job details*

The socio-demographic information including age, gender, educational level (graduate or post graduate) and marital status (single or married), exercise (no or yes) and smoking habits (no or yes) of participating nurses were recorded in the first part of the questionnaire. Job details included: job experience (years), work schedule (normal work day or 8 hours, 12 hours and 24 hours rotating shift) and job satisfaction (‘How much are you satisfied with your job? low, moderate, or high’).

*Fatigue*

The 20-item Multidimensional Fatigue Inventory (MFI–20)^14^ was used for evaluation of fatigue in the study. The MFI–20 is a valid and reliable tool, which has been widely used in previous research for evaluation of fatigue in both patients and healthy subjects.^15-17^This MFI–20 consists of 5 dimensions including: (1) general fatigue, (2) physical fatigue, (3) reduced motivation, (4) mental fatigue, and (5) reduced activity. Each dimension has four items and scored with a 5-point Likert-type format. The possible score for each dimension range from 4 to 20, with higher scores showing higher fatigue. The revised version of the MFI–20 in Persian language, with established validity and reliability, was used in this study.^18,19^ The Cronbach’s α for the MFI–20 and its dimensions in this study were as follows: total MFI = 0.86, general fatigue = 0.74, physical fatigue = 0.72, reduced activity = 0.70, reduced motivation = 0.76 and mental fatigue = 0.73.

*Psychological distress*

The 12-item General Health Questionnaire (GHQ–12),^20^ which is a well-tried and tested technique, was used for the evaluation of psychological distress among nurses. Each item in this tool is rated on a 4-point response format (0 = less than usual, 0 = no more than usual, 1 = rather more than usual, or 1 = much more than usual), and the total GHQ–12 score ranges from 0 to 12. In this study, the threshold for cases classification (e.g., poor psychological distress) was GHQ score of 4 or higher.^1^ The Farsi version of the GHQ–12, which has an established validity and reliability, was used in the study.^21^ The Cronbach’s α for the GHQ–12 in this study was 0.89.

### Data analysis

Statistical analysis of the data was carried out using SPSS software version 21 (IBM Inc., Armonk, NY, USA). The socio-demographic and job details of the participating nurses are presented were tabulated (as mean, standard deviation [SD], range and percentages). The internal consistency of the questionnaires was assessed using Cronbach’s α. Stepwise multiple linear regression analysis was performed to evaluate the rela­tionship between fatigue and psychological distress. R square coefficients were also calculated. Furthermore, error bar was used to show the relationship between the mean scores of psychological distress and MFI–20 dimensions. *P* values less than 0.05 were considered as statistically significant.

## Results

### Description of the study sample

Socio-demographic and job details of the study participating are presented in Table 1. Most participants were female (85.7%), married (75.5%) and had undergraduate education (95.4%). The majority of nurses reported that they regularly exercise (97.9%) are non-smokers (96.4%). The age and job experience of the participants ranged between 24–54 years (mean ± SD = 33.4 ± 6.1 years) and 1–32 years (mean ± SD = 9.1 ± 5.8 years), respectively. The percentage of nurses who reported low, moderate and high levels of job satisfaction were 24.7%, 59.1% and 18.2%, respectively.

### Fatigue and psychological distress

The scores of the MFI–20 for the study participants are presented in Table 2. The total fatigue score of the MFI was 52.5 (range = 20–100; SD = 11.8), with the general fatigue (mean ± SD = 13.2 ± 3.1) and reduced motivation (mean ± SD = 9.2 ± 2.7) being the dimensions with the highest and lowest mean scores, respectively.

The percentage of psychological distress in this study (e.g., those nurses scored above the cut-off point – GHQ–12 ≥ 4) was 29.1%.

### Relationship between fatigue and psychological distress 

The results of stepwise multiple linear regression analysis (as shown in Table 3), which evaluated the relationship between fatigue (MFI dimensions scores) and psychological distress, indicated that the physical fatigue, mental fatigue and general fatigue were remained in the model (*P* < 0.001), while reduced motivation and reduced activity were excluded from the model as shown below:

MFI = -3.59 + 021 (physical fatigue) + 0.22 (mental fatigue) + 0.17 (general fatigue) + 0.11 (reduced motivation)

**Figure 1:**
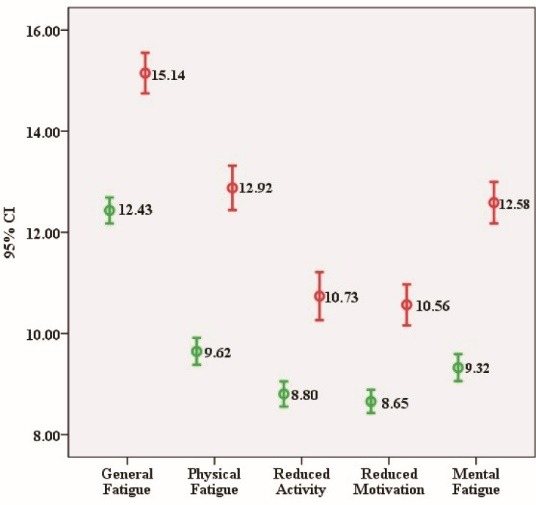
Relationship between psychological distress and MFI–20 dimensions scores.
Note: red bars indicate the scores of MFI dimensions for those nurses with a higher level of psychological distress (above the cut-off point) and green bars indicate the scores of MFI dimensions for those nurses with a lower level of psychological distress (below the cut-off point). The general, physical and mental fatigue dimensions were significantly different between two groups.

The scores of MFI dimensions were significantly higher for those nurses with a higher level of psychological distress (above the cut-off point) than those with a lower level of psychological distress (below the cut-off point) (Figure 1).

## Discussion

The aim of this study was to evaluate the relationship between different dimensions of fatigue and psychological distress among public hospital nurses. It was found that the fatigue and psychological distress were fairly common among the studied nurses, and that several dimensions of fatigue (including general, physical and mental fatigue) were related to the presence of psychological distress in this population. These findings may reveal the poor and stressful condition poor and stressful conditions within which the nursing personnel are expected to work.

The results of the present study confirm that fatigue is a common problem in hospital nursing personnel. This is one of the first studies which uses the MFI–20 for fatigue evaluation in nurses, and thus it is not possible to compare the findings directly. Nevertheless, our findings are in agreement with previous studies showing a high prevalence of fatigue in this working population.^4-7,22^ This is perhaps not surprising and can be attributed to a number of factors including high job demands and low control, shift working and skill discretion.^11,23-26^

As shown in this research, the physical and mental aspects of fatigue were found to be more problematic than other aspects such as reduced motivation and reduced activity in hospital nurses. This finding emphasizes the importance of both physical and mental aspects of fatigue in this group of healthcare professionals. The results of a study conducted by Barker and Nussbaum^22^ among nurses in the United States indicated that mental fatigue was more prevalent than physical fatigue in their subjects, which is not in agreement with our findings. This discrepancy can possibly be explained by different tool being used for valuation of fatigue in the previous study compared to the present study. Alternatively, it might be attributed to the working condition of hospital nurses in the present study which may differ from that of their counterparts in the United States. Nevertheless, our findings highlight that both mental and physical aspects of fatigue deserve further attention. Therefore, multi-component interventions aimed at decreasing both mental (e.g., the need for new skills to cope with advances in their job, role in quality of care, direct contact with patients, etc.) and physical (e.g., overexertion, repeated lifting and transferring of patients, awkward working postures, etc.) workload of this working population are recommended.

According to our findings, the GHQ–12 score was above the cut-off point for about one-third of the studied hospital nurses. This finding indicates that psychological distress is fairly common among this group of healthcare professionals and is generally in agreement with the findings reported in other countries. Su et al^13^ reported a higher prevalence of psychiatric disorders (42%) in nurses than in other hospital staff in Taiwan. Suzuki et al^1^ also reported a high prevalence of poor psychological distress (68.8%) among their study sample in Japan. These findings reveal the stressful working condition of hospital nurses and emphasize the need for ergonomic interventions (e.g., job organization and re-design, attempts to reduce mental demands, training programmes, etc.) to reduce the psychological distress among this working group.

With regard to the relationship between fatigue and psychological distress, our results confirmed a significant relationship between these two variables, so that those nurses with poor psychological distress (GHQ–12 ≥ 4) experienced a higher level of fatigue than those with better psychological distress (GHQ–12 < 4). According to the stepwise multiple linear regression model, physical and mental aspects of fatigue were significantly related to the psychological distress, while reduced motivation and reduced activity dimensions had no effect in this regard. This finding is of particular since with regard to the multidimensional nature of fatigue, the use of MFI–20 can provides a better understanding regarding the various dimensions of fatigue. This finding is relatively new to the literature and highlights the role of both physical and mental fatigue in the presence of psychological distress among hospital nurses. Therefore, interventions aiming at improving psychological distress in this working population should give more attention to these dimensions of fatigue.

The contributions of the present study should be viewed in light of several limitations. First, the data in this study were collected at a single time point, and therefore causality cannot be established. Thus, further studies may be required to replicate these results using prospective longitudinal data. Moreover, limitations related to the generalizability of the findings should also be considered. This is particularly the case with the response rate in this research, which may introduce a source of selection bias. Nevertheless, it should be pointed out that since the majority of nurses in hospitals or clinical settings experience or are exposed to relatively high job demands; the current findings may have practical implications in terms of reducing fatigue and improving psychological distress in this population in different work settings.

## Conclusion

In line with findings from previous research, the results of this study revealed that fatigue and psychological distress were fairly prevalent in the studied hospital nurses. The results showed significant relationship between several dimensions of fatigue (particularly physical and mental dimensions) and psychological distress in this group of healthcare professionals. These findings emphasize the need for interventions aimed at decreasing both mental and physical fatigue as they have potential to influence the psychological distress in this working population.

## Ethical approval

The study protocol was approved by the ethics committee of the Tabriz University of Medical Sciences.

## Competing interests

There are no conflicts of interest.

## Authors’ contributions

AB involved in the conceptualization and designing of the study. ID involved in the data analysis and drafting the manuscript. LR and HZ involved in the data collection.

## Acknowledgements

The authors wish to acknowledge the support and assistance provided by the studied hospitals, and all the principals who collaborated in this study.

**Table 1:** Demographic and job characteristics of the study population (n = 699)

**Variables**	
Gender (%)	
Male	14.3
Female	85.7
Age (y)	
Mean ± SD	33.4 ± 6.1
Range	24–54
BMI (kg/m^2^)	
Mean ± SD	24.7 ± 3.2
Range	15.9–37.5
Marital status (%)	
Single	24.5
Married	75.5
Education level (%)	
Undergraduate	95.4
Post graduate	4.6
Regularly exercise (%)	
Yes	97.9
No	2.1
Smoking (%)	
Yes	3.6
No	96.4
Job experience (y)	
Mean ± SD	9.1 ± 5.8
Range	1–32
Work schedule (%)	
Shift work	75.8
Work day	27.7
Job satisfaction (%)	
Low	24.7
Moderate	59.1
High	18.2

**Table 2:** MFI–20 scores

**Variables**	**Mean**	**SD**	**Min**	**Max**
General fatigue	13.2	3.1	4	20
Physical fatigue	10.5	3.3	4	20
Reduced activity	9.3	3.0	4	20
Reduced motivation	9.2	2.7	4	20
Mental fatigue	10.2	3.3	4	20
Total MFI	52.5	11.8	20	100

**Table 3:** Relationship between psychological distress and fatigue (MFI–20 dimensions scores) based on the stepwise multiple linear regression analysis

**Variables retained in the model**	**B**	**SE**	**Standard β**	***P*** **value**
Physical fatigue	0.21	0.04	0.22	<0.001
Mental fatigue	0.22	0.04	0.23	<0.001
General fatigue	0.17	0.03	0.18	<0.001
Constant	-5.33	0.47	–	<0.001
